# Multistimuli Sensitive Behavior of Novel Bodipy‐Involved Pillar[5]arene‐Based Fluorescent [2]Rotaxane and Its Supramolecular Gel

**DOI:** 10.1002/advs.201500082

**Published:** 2015-05-26

**Authors:** Nana Sun, Xin Xiao, Wenjun Li, Jianzhuang Jiang

**Affiliations:** ^1^ Beijing Key Laboratory for Science and Application of Functional Molecular and Crystalline MaterialsDepartment of ChemistryUniversity of Science and Technology BeijingBeijing100083China; ^2^ Department of ChemistryGuizhou UniversityGuiyang550025China

**Keywords:** Bodipy, fluorescence, pillar[*n*]arene, rotaxane, supramolecular gels

## Abstract

Fluorescent [2]rotaxane BC12P5 is successfully constructed with 1,4‐diethoxypillar[5]arene as wheel over a long alkyl axle with Bodipy chromophore as one stopper for the first time. NMR spectra clearly reveal its molecular shuttle nature triggered by multiple external stimuli including solvent polarity and temperature. In particular, the fluorescence nature introduced into [2]rotaxane BC12P5 renders it a good sensor for the external stimuli. Nevertheless, the supramolecular gel successfully fabricated from this novel rotaxane system via self‐assembly in dimethyl sulfoxide (DMSO) also shows reversible gel–sol phase transition upon multiple external stimuli such as heating/cooling, shaking/resting, or the addition of different anions. Interestingly, exposure of the supramolecular gel film to HCl or ammonia vapor induces the change in the film fluorescence intensity, endowing this system with a potential application in gas detecting.

## 1 Introduction

This is an open access article under the terms of the Creative Commons Attribution License, which permits use, distribution and reproduction in any medium, provided the original work is properly cited.

Inspired by naturally occurring biological motors such as the ATPase rotary motor and the kinesin or myosin linear motor systems,^1^ chemists have tried to construct a variety of artificial molecular machines^2^ including the unidirectional rotors, shuttles, scissors, and molecular muscles that can perform diverse molecular motions.^3^ Rotaxanes, with a typical mechanically interlocked structure, have been widely employed as crucial precursor and building blocks for the fabrication of advanced supramolecular architectures^4^ like molecular shuttles and switches with molecular motions upon certain external stimuli.^5^ Thus far a series of macrocyclic compounds including crown ethers, cyclodextrins, and cucurbit[*n*]urils have been applied to construct the rotaxane‐based molecular motors.^6^ Pillar[*n*]arenas, as the new member of functional macrocyclic family, have also been employed as good building block to create rotaxanes shortly after their first synthesis in 2008.^7^ In 2011, Stoddart and co‐worker reported the first pillar[5]arene‐based [2]rotaxane formed between a pillar[5]arene and *N*,*N*′‐bis(3,5‐di‐*tert*‐butylbenzyl)octane‐1,8‐diamine,^8^ which was followed by the construction of different species of [2]rotaxanes^9^ and novel [3]rotaxanes.^10^ However, pillararene‐based rotaxanes with multiple external stimuli responsiveness, especially those incorporating fluorogenic functionalities remain rarely explored.

On the other hand, boron dipyrromethene (Bodipy) dyes have constituted one of the most important families of simple organic luminophores due to their special absorption and emission properties.^11^ Their strong absorption and emission in the visible and near‐infrared range render them great application potential in chemosensors and probes, biological labels, laser dyes, photodynamic therapy agents, and a plethora of photonic devices.^12^ Bodipy‐involved rotaxanes have, however, been rarely reported thus far, limited to several crown ether/cucurbit[*n*]uril‐based rotaxanes,^13^ to the best of our knowledge.

**Scheme 1:**
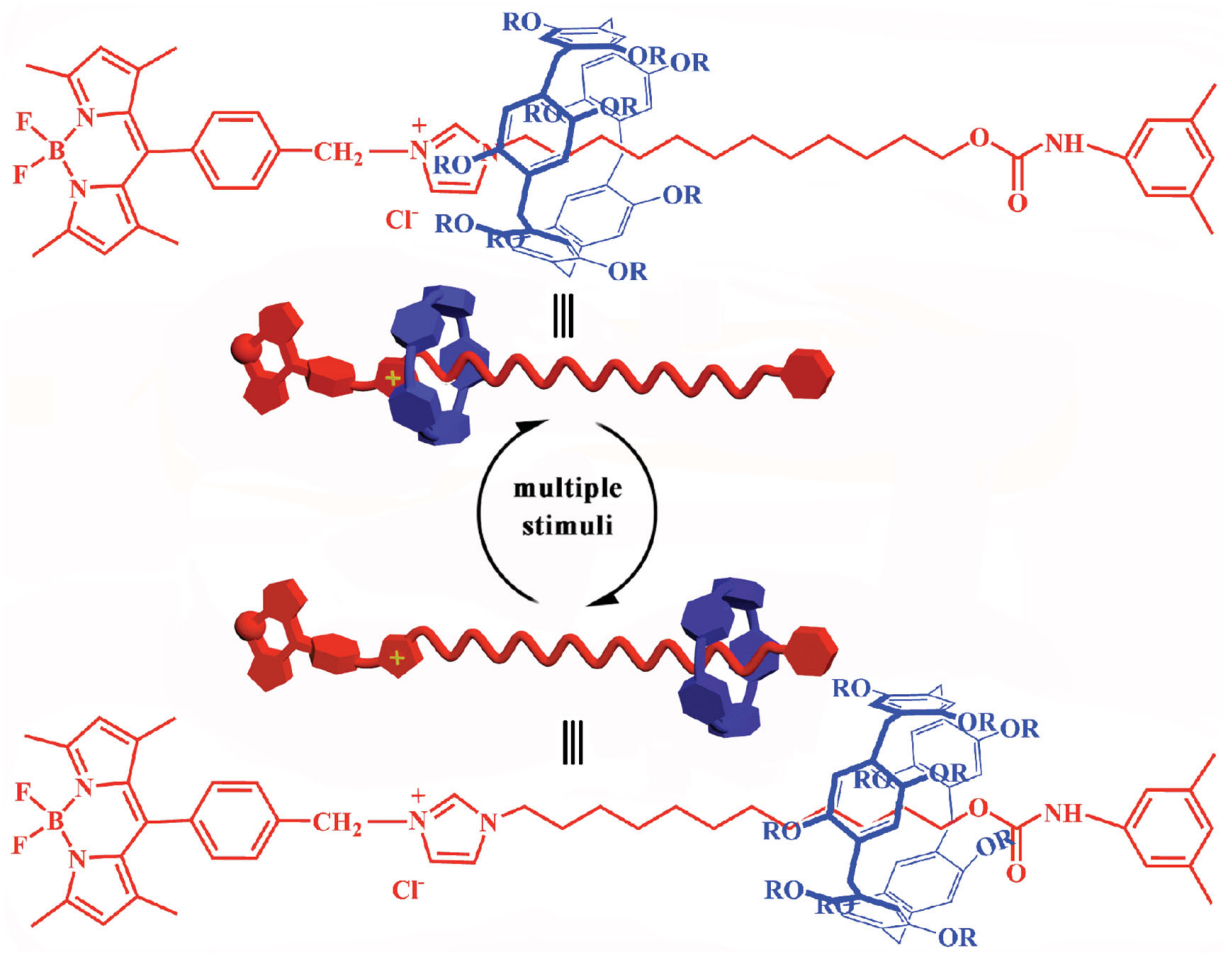
Schematic molecular structures of [2]rotaxane BC12P5 under external stimuli.

In the present paper, we describe the preparation and characterization of a new type of fluorescent [2]rotaxane BC12P5 constructed on the basis of pillar[5]arene wheel and a dumbbell‐shaped axle with Bodipy chromophore as one stopper, **Scheme** **1**, which appears to represent the first Bodipy‐involved pillar[5]arene‐based rotaxane. The pillar[5]arene wheel of this novel [2]rotaxane BC12P5 was revealed to move over its dumbbell‐shaped alkyl axle under multiple external stimuli including the solvent polarity, temperature, and pH value on the basis of the NMR and fluorescent spectroscopic investigations. Nevertheless, [2]rotaxane BC12P5 is able to self‐assemble into supramolecular gel in dimethyl sulfoxide (DMSO), which also shows reversible gel–sol phase transition upon multiple external stimuli like heating/cooling, shaking/resting, or the addition of different anions.

At the end of this section, it is noteworthy that in the past decade supramolecular gels formed by self‐assembly of organic molecules into entangled structures to immobilize the solvents have attracted extensive research interests due to their potential applications in chemosensors, optoelectronic devices, drug delivery, tissue engineering, biomaterials, and surface science.^14^ The present Bodipy‐containing pillar[5]arene‐based fluorescent gel is therefore expected to find applications in related fields.

## 2 Results and Discussion

### 2.1 Molecular Design and Synthesis

Generally, rotaxane is constructed by wheel and axle components. In the present case, 1,4‐diethoxypillar[5]arene (EtP5) is chosen as the wheel of the target [2]rotaxane BC12P5 with 12‐(1H‐imidazol‐1‐yl)dodecanol (**1**) stopped by a carbamic unit and a meso‐chloro‐benzyl‐Bodipy unit (**2**) at both ends as an axle, Scheme 1. Interestingly, the Bodipy unit introduced as one stopper of the axle also provides the [2]rotaxane BC12P5 with an effective fluorescence chromophore, enabling the detection of the responsiveness to external stimuli by fluorescence method. Both EtP5 and the semiblocked rod‐like Bodipy derivative Bodipy‐C12OH (**3**) were prepared according to the published procedures.[[qv: 7d]],[[qv: 12b]] Reaction of EtP5 with the semiblocked rod‐like component Bodipy derivative **3** in CHCl_3_ led to the formation of pseudorotaxane structure, which then reacted with 1‐isocyanato‐3,5‐dimethylbenzene in CHCl_3_ afforded the target [2]rotaxane BC12P5. Satisfactory elemental analysis result was obtained for the newly prepared [2]rotaxane BC12P5 after repeated column chromatography followed by recrystallization. The matrix‐assisted laser desorption/ionization time of flight (MALDI‐TOF) mass spectrum displayed intense signal at *m*/*z* = 1627.95, corresponding to the molecular ion [M–Cl]^+^. Nevertheless, the key intermediate and the target [2]rotaxane BC12P5 were also characterized by ^1^H and ^13^C NMR spectroscopies, Figures S1–S6 (Supporting Information).

### 2.2 NMR Characterization

**Figure 1:**
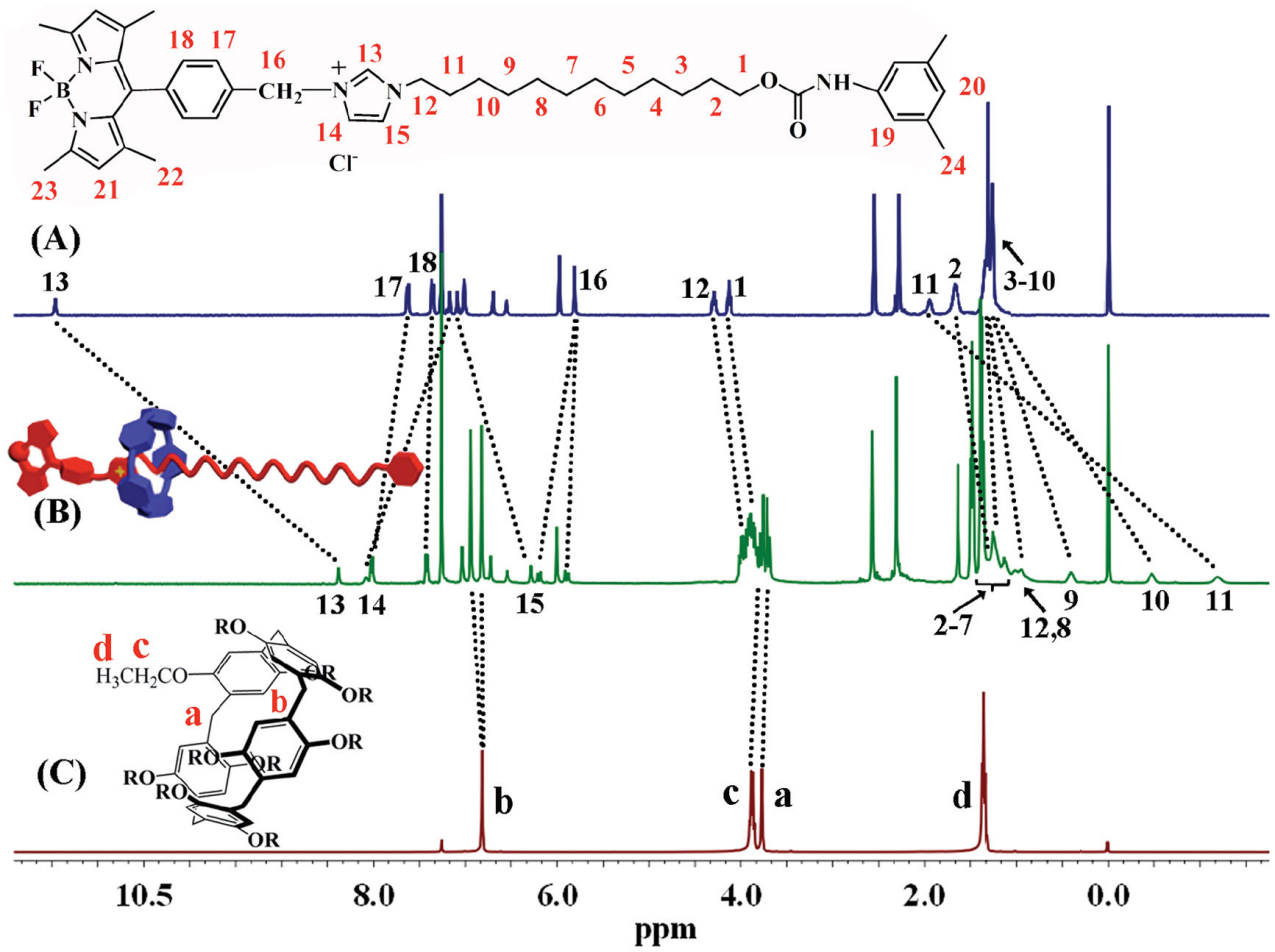
^1^H NMR spectra of A) compound **4**, B) [2]rotaxane BC12P5, and C) EtP5 recorded in CDCl_3_ at 25 °C.

**Figure** **1** and Figure S7 (Supporting Information) show the ^1^H NMR and 2D nuclear Overhauser enhancement spectroscopy (NOESY) of [2]rotaxane BC12P5 in CDCl_3_. For comparative study, the NMR spectra for the two components, namely the wheel and the axle Bodipy‐C12OH‐isocyanato (**4**), in CDCl_3_ were also recorded and shown in Figure 1. Comparison in the NMR spectrum of these three species reveals that the ^1^H NMR spectrum of [2]rotaxane BC12P5 is not a simple superimposition of the spectra of pure compound **4** and EtP5 in the same deuterated solvent, indicating the effective interaction between the host wheel and the guest axle in the supramolecular rotaxane system. As can be seen, Figure 1 and Table S1 (Supporting Information), after being fabricated into [2]rotaxane BC12P5, the signals of the methylene protons H_9_, H_10_, H_11_, and H_12_ on the axle (which are adjacent to the imidazolium unit in the axle) take obvious upfield shift from 1.31, 1.31, 1.96, 4.29 to 0.39, −046, −1.19, and 3.98 ppm, respectively. This is also true for the imidazolium protons H_13_ and H_15_ with substantial upfield shift from 11.47 and 7.09 to 8.39 and 6.30 ppm, respectively. These results suggest the encapsulation of the imidazolium moiety and its adjacent methylene groups in the axle by the pillar[5]arene wheel in [2]rotaxane BC12P5 in CDCl_3_. Additional support for this point comes from the cross‐peaks between the aromatic protons H_b_ of pillar[5]arene ring and the methylene protons H_9_ and H_10_ observed in the NOESY spectrum of [2]rotaxane BC12P5, Figure S7 (Supporting Information).

To reveal the solvent polarity effect on the [2]rotaxane BC12P5 conformation, a more polar solvent, DMSO‐*d*_6_, was utilized for comparative NMR investigations. As shown in Figure S8 and Table S2 (Supporting Information), the signals of the methylene protons H_1_, H_2_, H_3_, H_4_, H_5_, and H_6_ that are adjacent to the carbamic stopper in the axle exhibit substantial upfield shift (Δ*δ* = −0.38, −0.84, −1.67, −2.08, −1.91, and −1.47 ppm, respectively) in the rotaxane system in comparison with those for pure compound **4**, revealing the shielding effect of the host EtP5 cavity on these protons and in turn suggesting the methylene groups adjacent to the carbamic stopper in the axle threaded into the cavity of the pillar[5]arene ring in [2]rotaxane BC12P5 in DMSO‐*d*_6_. This is further confirmed by the cross‐peaks between the signals of methylene protons H_3_, H_4_, H_5_, and H_6_ of the alkyl chain and the phenyl proton H_b_ of the pillar[5]arene moiety observed in the 2D NOESY spectrum of [2]rotaxane BC12P5 in DMSO‐*d*_6_, Figure S7 (Supporting Information). Obviously, different conformation was employed by [2]rotaxane BC12P5 in DMSO‐*d*_6_ from that in CDCl_3_ due to the difference in the solvent and [2]rotaxane BC12P5 intermolecular interactions, suggesting the possible solvent polarity‐driven molecular shuttle nature of this system.

### 2.3 Solvent Polarity‐Driven Molecular Shuttle

**Figure 2:**
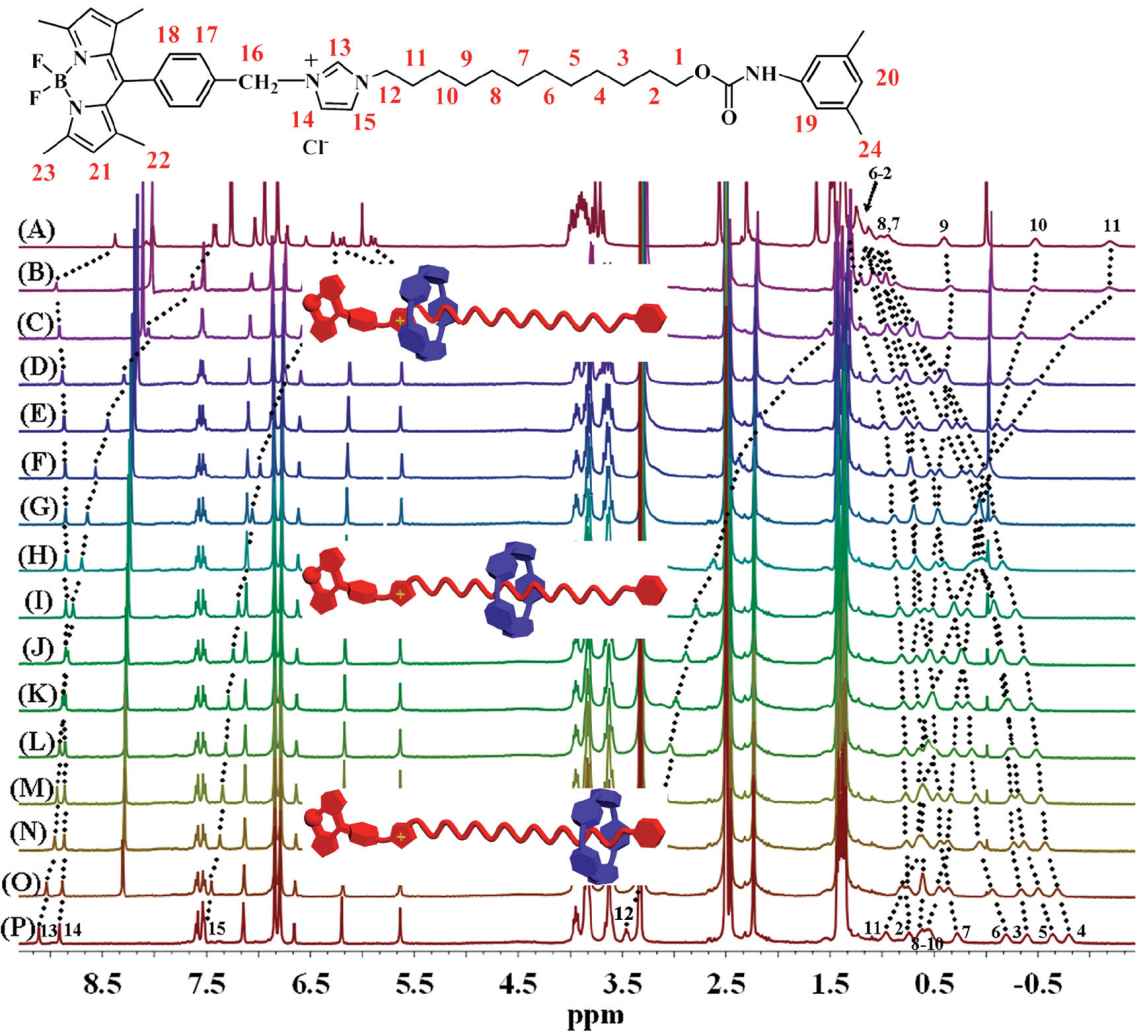
Systematic change in the ^1^H NMR spectrum of [2]rotaxane BC12P5 along with the change in the ratio of CDCl_3_/DMSO‐*d*_6_ (v/v): A) CDCl_3_, B) 1:1, C) 1:1.5, D) 1:2, E) 1:2.5, F) 1:3, G) 1:3.5, H) 1:4, I) 1:5, J) 1:6, K) 1:7, L) 1:8, M) 1:9, N) 1:10, O) 1:20, and P) DMSO‐*d*_6_ recorded at 25 °C.

As described above, ^1^H NMR measurements indicate that the solvent polarity change might be able to induce the pillar[5]arene cavity to move on the alkyl chain in [2]rotaxane BC12P5. As a result, systematic studies over the ^1^H NMR spectra of [2]rotaxane BC12P5 in a series of mixed solvents with different ratio of CDCl_3_/DMSO‐*d*_6_ were carried out. As shown in **Figure** **2** and Table S3 (Supporting Information), along with the decrease in the solvent polarity due to the stepwise addition of CDCl_3_ into DMSO‐*d*_6_, the signals of the methylene protons H_9_, H_10_, H_11_, and H_12_ and the imidazolium protons H_13_ in the axle of [2]rotaxane BC12P5 experience substantial upfield shift. However, the signals of the methylene protons (that are adjacent to the carbamic stopper) such as H_2_, H_3_, H_4_, H_5_, and H_6_ take obvious downfield shift, demonstrating the gradual movement of the pillar[5]arene moiety on the axle from the methylene groups adjacent to the carbamic stopper to those adjacent to the imidazolium unit along with the decrease in the solvent polarity, revealing the solvent polarity‐driven molecular shuttle nature of [2]rotaxane BC12P5.

### 2.4 Thermodriven Molecular Shuttle

In order to try to investigate the molecular motion of [2]rotaxane BC12P5 under temperature stimuli, the temperature‐dependent ^1^H NMR spectra for the rotaxane system were recorded in DMSO‐*d*_6_. As exhibited in Figure S9 and Table S4 (Supporting Information), along with increasing the temperature, the signals of the methylene protons H_9_, H_10_, H_11_, and H_12_ (which are close to the Bodipy stopper) and the imidazolium protons H_13_, H_14_, and H_15_ gradually move to upfield direction. For instance, the signal of proton H_12_ shifts from 3.46 to 2.19 ppm along with the temperature change from 25 to 115 °C. In contrast, the signals of methylene protons H_2_, H_3_, H_4_, H_5_, and H_6_ (which are adjacent to the carbamic stopper) gradually move to the downfield direction as the temperature increases as exemplified by the shift of proton H_2_ signal from 0.75 ppm at 25 °C to 1.10 ppm at 115 °C, Figure S10 and Table S4 (Supporting Information). These results clearly reveal the temperature‐driven molecular shuttle nature of [2]rotaxane BC12P5. Nevertheless, on the basis of the just above section and according to these NMR spectroscopic results, the pillar[5]arene ring[2]rotaxane BC12P5 should locate on the methylene groups of the axle that are adjacent to the carbamic stopper at low temperature in DMSO‐*d*_6_. Along with increasing the temperature, the pillararene ring gradually slides to the imidazolium unit.

### 2.5 Fluorescence Properties of [2]rotaxane BC12P5 in Solution

#### 2.5.1 Effects of Solvent Polarity on the Fluorescence Properties

Due to the incorporation of the Bodipy fluorescent chromophore in the present rotaxane system, the fluorescence properties of this system were therefore studied following the solvent polarity change. As displayed in Figure S11 (Supporting Information), with the system concentration being fixed at 1 × 10^−5^ m, the fluorescence intensity of [2]rotaxane BC12P5 gradually gets decreased along with the addition of CHCl_3_ into the solution of DMSO. In pure CHCl_3_, a total decrease by the most of 23% in the fluorescence intensity was achieved in comparison with that in pure DMSO. In good contrast, the fluorescence property of compound **4** was also studied. As shown in Figure S11 (Supporting Information), the fluorescence intensity of **4** gradually gets decreased along with the addition of CHCl_3_ into the solution of DMSO. In pure CHCl_3_, the fluorescence intensity of compound **4** decreases for about 37% in comparison with that in pure DMSO. This is in line with that observed for [2]rotaxane BC12P5. However, the decrease in the fluorescence intensity for compound **4** is larger than that of [2]rotaxane BC12P5. This seems to indicate the relatively less effect of the solvent polarity‐driven molecular shuttle motion on the fluorescence intensity, suggesting the more effect of the solvent polarity on the fluorescence intensity. In line with previous investigation,^15^ in the present case higher fluorescence intensity for [2]rotaxane BC12P5 in polar solvent is achieved due mainly to the decrease in the nonradiative rate constant (which minimizes the nonradiative energy loss) with the help of the solvent polarity‐driven molecular shuttle motion.

#### 2.5.2 Effect of Temperature on the Fluorescence Properties

**Figure 3:**
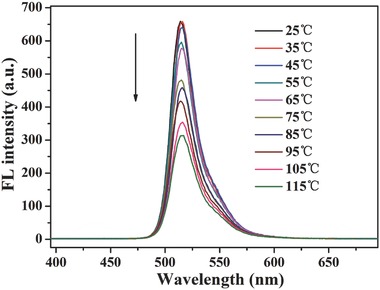
Systematic change in the fluorescence spectrum of [2]rotaxane BC12P5 in DMSO (1 × 10^−5^ mol L^−1^) along with the temperature change from 25 to 115 °C.

As can be easily expected, the fluorescence intensity of [2]rotaxane BC12P5 at a fixed concentration of 1 × 10^−5^ m in DMSO also takes systematic change along with the change in temperature, gradually decreased along with the temperature increase, by the most of 52% at 25 °C in comparison with that at 115 °C, **Figure** **3**. This is also true for the reference compound **4**. As shown in Figure S12 (Supporting Information), along with increasing the temperature, the fluorescence intensity of **4** gets gradually decreased in a similar manner to that of [2]rotaxane BC12P5, indicating the weaker influence of the thermodriven molecular shuttle movement of [2]rotaxane BC12P5 on the axle to the fluorescence intensity than that due to the consumption of more nonradiative energy at high temperature.^16^

#### 2.5.3 Effect of Acid/Base Change on the Fluorescence Properties

**Figure 4:**
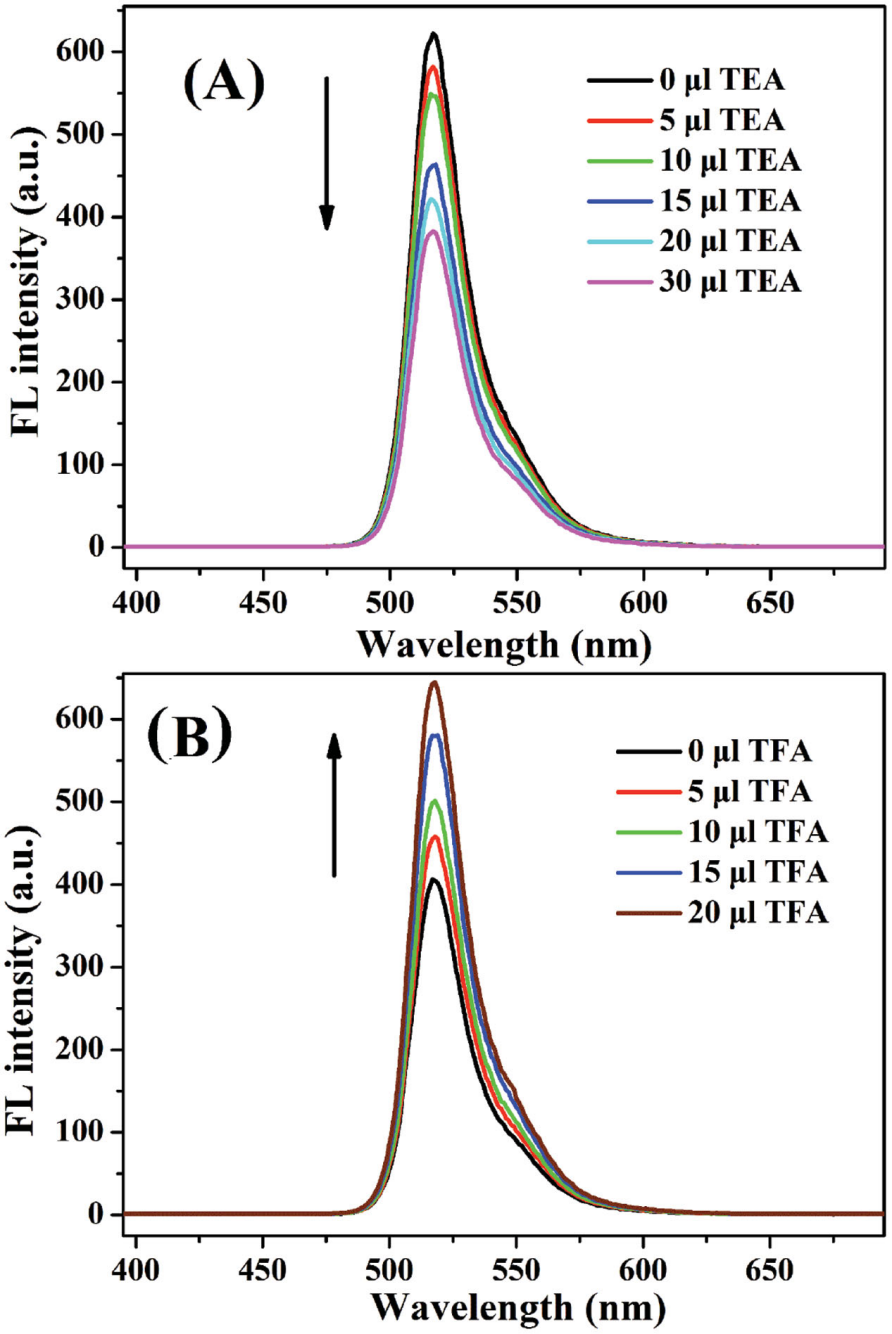
The fluorescence emission spectra of [2]rotaxane BC12P5 (1 × 10^−5^ mol L^−1^) in CHCl_3_ upon addition of increasing amount (0, 5, 10, 15, 20, and 30 μL) of TEA (A) and then of increasing amount (0, 5, 10, 15, and 20 μL) of TFA at 25 °C (B).

Acid/base titration experiments were also carried out to reveal the sensor property of [2]rotaxane BC12P5. As can be seen in **Figure** **4**, along with the gradual addition of triethylamine (TEA) into the CHCl_3_ solution of [2]rotaxane BC12P5 from 0 to 30 μL, the fluorescence intensity at 516 nm undergoes a consecutive decrease of 38%. However, the fluorescence spectrum could completely recover upon addition of trifluoroacetic acid (TFA) into the above‐described CHCl_3_ solution from 0 to 20 μL, indicating the influence of the pH on the fluorescence intensity. On the basis of previous research result,^17^ the fluorescence‐quenching photoinduced electron transfer (PET) between the amine and Bodipy core at the extreme of high pH is able to occur because electron transfer may occur through space, resulting in the recovery of the fluorescence intensity of [2]rotaxane BC12P5 due to the neutralization upon addition of TFA. As can be expected, the fluorescence intensity for **4** upon addition of TEA into the solution also gets decreased in quite a similar manner to that of [2]rotaxane BC12P5, Figure S13 (Supporting Information). Nevertheless, the fluorescence intensity for this system also gets recovered after adding TFA.

### 2.6 Preparation of [2]rotaxane BC12P5 Supramolecular Gel

**Scheme 2:**
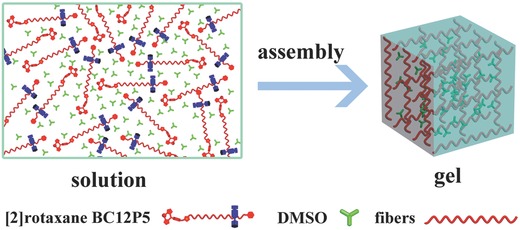
Schematic representation of self‐assembling [2]rotaxane BC12P5 into the supramolecular gel via 1D aggregate in DMSO.

**Figure 5:**
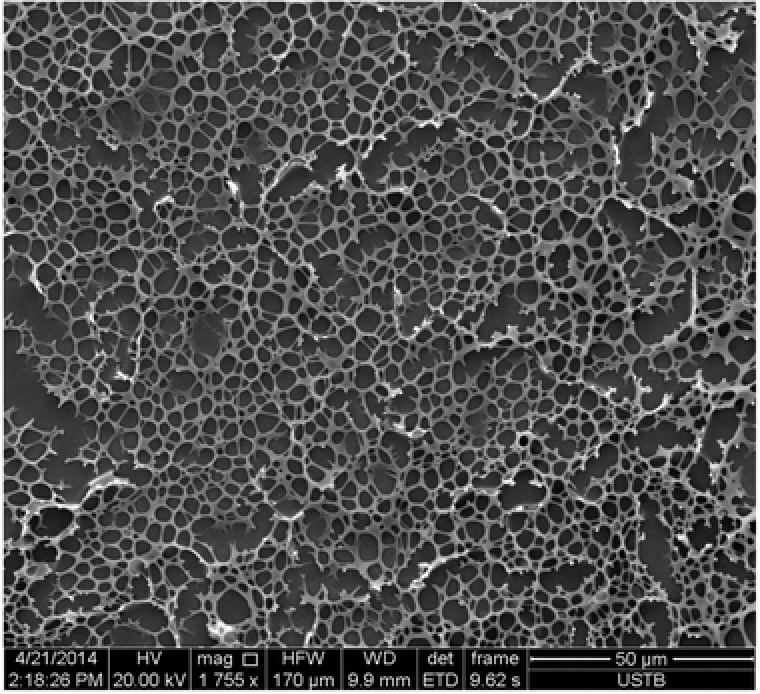
SEM image of the supramolecular gel of [2]rotaxane BC12P5 formed in DMSO by drop casting on the copper grid.

Supramolecular gels constructed from low‐molecular‐weight molecules (LMWMs) simultaneously possessing both toughness and flexibility as gelators depending on reversible noncovalent interactions play important role in the development of soft material science.[[qv: 14d]] Due to the relatively rigid structure of the host component and the soft structure of the guest component usually employed by the rotaxanes and pseudorotaxanes, either rotaxanes or pseudorotaxanes with a mechanically interlocked structure have been used as suitable gelators to construct supramolecular gels.^18^ In the present case, the π–π interactions between the phenylene moieties of neighboring pillar[5]arene rings of [2]rotaxane BC12P5, as well as between the Bodipy–Bodipy stacking, Bodipy, and imidazolium moieties, with the help of the van der Waals forces between long alkyl chains in the neighboring [2]rotaxane BC12P5 systems result in the formation of 1D supramolecular arrays, which subsequently self‐assemble into the cross‐linked network depending on the similar intermolecular interactions as mentioned above between neighboring [2]rotaxane BC12P5 systems in different 1D arrays, **Scheme** **2**. This 3D network then entraps the DMSO molecules with its porous structure, leading to the formation of a novel supramolecular gel containing Bodipy fluorescence chromophore depending mainly on the hydrogen bonding interaction between the solvent DMSO molecules and [2]rotaxane BC12P5 systems.^19^ The critical gelation concentration for [2]rotaxane BC12P5/DMSO was about 11.2 wt%. **Figure** **5** shows the scanning electron microscope (SEM) image of the gel formed from [2]rotaxane BC12P5. The 3D network constructed from nanofibers with an interconnected porous structure observed clearly reveals the gel nature of this system.

### 2.7 Multiple Stimuli‐Responsive Reversible Gel–Sol Transitions of the Supramolecular Gel

**Figure 6:**
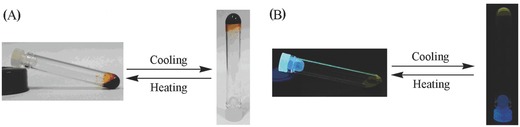
Photographs of reversible sol–gel transition upon cooling/heating under A) ambient light and B) illuminated at 365 nm.

Due to the sensitivity of the noncovalent interactions to external stimuli including solvent, temperature, pH, and mechanical stress, supramolecular gels usually exhibit stimuli responsiveness to the environment. As a consequence, multiple stimuli‐responsive behaviors of the present supramolecular gels were also investigated. Similar to other gels, a reversible gel–sol transition could be easily achieved by shaking (here through ultrasonic waves) or resting of this gel system. In addition, after adding CF_3_COOAg into the supramolecular gel system, the gel gradually collapses and finally becomes a solution after removing the AgCl precipitate. Upon addition of a little excess amount of tetrabutylammonium chloride (TBACl) into the solution, supramolecular gel is reformed.[[qv: 5j]],^20^ Nevertheless, most probably associated with the temperature‐dependent nature of its building block, the supramolecular gel fabricated from [2]rotaxane BC12P5 is also sensitive to temperature. As displayed in **Figure** **6**, along with increasing the temperature, the supramolecular gel gradually becomes a solution with the gel‐to‐solution phase transition temperature (*T*_gel_) of about 338 K. Reversibly, along with decreasing the temperature, the solution formed over 338 K return to the gel phase.

This gel also shows acid/base stimuli‐responsiveness. In comparison with [2]rotaxane BC12P5 in DMSO solution, the supramolecular gel thin film prepared by coating onto quartz slide shows a redshifted broadened fluorescence at 534 nm with obviously weakened intensity due to the enhanced intermolecular interactions of [2]rotaxane BC12P5 in the gel state, Figure S14 (Supporting Information). Interestingly, upon being exposed to HCl gas, the thin film gel fluorescence intensity gets significantly decreased, by the most of 50%, accompanied also by a visually clear fluorescence color change from yellow to purplish red under UV light, Figure S15 (Supporting Information). In contrast, when being exposed to the NH_3_ gas, the fluorescence intensity of the supramolecular gel thin film gets increased by the most of 100% but without showing the visually color change behavior. These results seem to suggest the potential of this supramolecular gel as the acidic/basic gas sensor.

## 3 Conclusion

In summary, the first Bodipy‐involved fluorescent [2]rotaxane BC12P5 was designed and prepared. This novel [2]rotaxane BC12P5 system exhibits molecular shuttle nature under multi­ple external stimuli including solvent polarity and temperature according to NMR spectra. In particular, the fluorescent nature introduced into [2]rotaxane BC12P5 renders it a good sensor for these external stimuli. Nevertheless, the self‐assembled supramolecular gel formed from this rotaxane system with the help of DMSO also shows multiple external stimuli‐induced reversible gel–sol phase transition upon shaking/resting, heating/cooling, or the addition of different anions. In particular, exposure of this supramolecular gel film to the HCl gas leads to obvious decrease in the fluorescence intensity accompanied by a visually clear fluorescence color change under UV light, endowing the system with a application potential in acidic gas detecting.

## 4 Experimental Section

*General Remarks*: All reagents were obtained from commercial sources without further purification. The compounds of **1**, **2**, **3**, **4**, and EtP5 were prepared according to the literature procedure.[[qv: 7d]],[[qv: 12b]]

*Measurements*: NMR spectra were recorded on a Bruker DPX 400 spectrometer in CDCl_3_ and DMSO‐*d*_6_. Electronic absorption spectra were recorded on a Hitachi U‐4100 spectrophotometer. Steady‐state fluorescence spectra were performed on an F4500 (Hitachi). MALDI‐TOF mass spectra was taken on a Bruker BIFLEX III ultra‐high resolution Fourier transform ion cyclotron resonance mass spectrometer with α‐cyano‐4‐hydroxycinnamic acid as matrix. Elemental analysis was performed on an Elementar Vavio El III.

*Preparation of 12‐(1H‐Imidazol‐1‐yl)dodecanol (**1**)*: 1H‐imidazole (6.80 g, 0.10 mol), NaOH (4.00 g, 0.10 mol), and 12‐bromododecan‐1‐ol (2.65 g, 0.10 mol) in DMSO (20 mL) were stirred at 70 °C for 24 h. The solvent was poured into water. After filtration, the residue was dried by air to give **1** as a white solid with 80% yield. ^1^H NMR (400 MHz, CDCl_3_, 25 °C, δ): 7.48 (s, 1H), 7.07 (s, 1H), 6.92 (s, 1H), 3.94 (t, *J* = 12 Hz, 2H), 3.66 (s, *J* = 12 Hz, 2H), 1.77 (m, 4H), 1.28 (m, 16H); ^13^C NMR (400 MHz, CDCl_3_, 25 °C, δ): 137.21, 129.52, 118.90, 63.15, 47.18, 36.06, 32.96, 31.19, 29.93, 29.65, 29.56, 29.54, 29.51, 29.49, 29.13, 27.36, 26.64, 25.87, 25.69. MS *m*/*z*: [M+K]^+^ calcd for C_15_H_28_N_2_O, 252.39; found, 291.75. Anal. calcd for C_15_H_28_N_2_O: C 71.38, H 11.18, N 11.10; found: C 71.29, H 11.23, N 11.21.

*Preparation of Meso‐chloro‐benzyl‐Bodipy (**2**)*: 4‐(Chloromethyl)benzoyl chloride (3.84 g, 20.3 mmol) was added dropwise to a stirred solution of 2,4‐dimethyl‐1H‐pyrrole (3.86 g, 40.6 mmol) in dichloromethane (200 mL) at room temperature under nitrogen, and the mixture was heated at 50 °C with stirring for 2 h. After vacuum evaporation of the solvent, toluene (150 mL), dichloromethane (15 mL), and triethylamine (13 mL) were added to the residual solid. The mixture was stirred at room temperature for 30 min under nitrogen and boron trifluoride diethyl etherate (18 mL) was then added. After heating at 50 °C for 1.5 h, the solvent was removed under vacuum. The crude product was purified by silica gel column chromatography with hexane/ethyl acetate (5/1, v/v) eluent to give **2** with 45% yield. ^1^H NMR (400 MHz, CDCl_3_, 25 °C, δ): 7.53 (d, *J* = 8 Hz, 2H), 7.29 (d, *J* = 8 Hz, 2H), 5.98 (s, 2H), 7.03 (s, 4H), 4.66 (s, 2H), 2.55 (s, 6H), 1.38 (s, 6H); ^13^C NMR (400 MHz, CDCl_3_, 25 °C, δ): 155.84, 143.17, 141.09, 138.77, 135.28, 131.49, 129.38, 128.61, 121.49, 45.73, 14.73. MS *m*/*z*: [M^+^] calcd for C_20_H_20_BClF_2_N_2_, 372.65; found, 372.15. Anal. calcd for C_20_H_20_BClF_2_N_2_: C 64.46, H 5.41, N 7.52; found: C 64.38, H 5.46, N 7.64.

*Preparation of Bodipy‐C12OH (**3**)*: **1** (0.25 g, 1.0 mmol) and **2** (0.37 g, 1.0 mmol) were refluxed in CH_3_CN (100 mL) for 7 d. After filtration and solvent evaporation, the crude product was precipitated by diethyl ether to yield compound **3** as a red solid with 67% yield. ^1^H NMR (400 MHz, CDCl_3_, 25 °C, δ): 11.41 (s, 1H), 7.64 (d, *J* = 8 Hz, 2H), 7.37 (d, *J* = 8 Hz, 2H), 7.18 (s, 1H), 7.10 (s, 1H), 5.98 (s, 2H), 5.81 (s, 2H), 4.32 (t, *J* = 16 Hz, 2H), 3.65 (t, *J* = 16 Hz, 2H), 2.55 (s, 6H), 1.95 (m, 2H), 1.57 (m, 2H), 1.32 (m, 22H); ^13^C NMR (400 MHz, CDCl_3_, 25 °C, δ): 156.14, 142.81, 140.25, 139.05, 136.64, 134.42, 131.31, 129.94, 129.50, 121.76, 121.65, 121.12, 63.03, 53.17, 50.59, 32.90, 30.29, 29.49, 29.38, 29.34, 29.30, 28.93, 26.34, 25.81, 14.74, 14.60. MS *m*/*z*: [M–Cl]^+^ calcd for C_35_H_48_BF_2_N_4_OCl, 589.59; found, 589.37. Anal. calcd for C_35_H_48_BF_2_N_4_OCl: C 67.26, H 7.74, N 8.96; found: C 67.33, H 7.66, N 8.89.

*Preparation of Bodipy‐C12OH‐isocyanato (**4**)*: A mixture of **3** (0.31 g, 0.5 mmol), dibutyltindilaurate (one drop), and 1‐isocyanato‐3,5‐dimethylbenzene (0.22 g, 1.5 mmol) in CHCl_3_ (0.3 mL) was stirred at −6 °C for 24 h. After filtration and solvent evaporation, the crude product was purified by flash column chromatography with (CH_2_Cl_2_/MeOH, 15:1, v/v) as eluent to yield compound **4** as a red solid with 76% yield. ^1^H NMR (400 MHz, CDCl_3_, 25 °C, δ): 11.47 (s, 1H), 7.64 (d, *J* = 8 Hz, 2H), 7.37 (s, *J* = 8 Hz, 2H), 7.17 (s, 1H), 7.09 (s, 1H), 7.01 (s, 2H), 6.70 (s, 1H), 6.55 (s, 1H), 5.98 (s, 2H), 5.81 (s, 2H), 4.32 (t, *J* = 16 Hz, 2H), 4.15 (t, *J* = 16 Hz, 2H), 2.55 (s, 6H), 2.28 (s, 6H), 1.94 (m, 2H), 1.62 (m, 2H), 1.34 (m, 22H); ^13^C NMR (400 MHz, CDCl_3_, 25 °C, δ): 156.16, 142.80, 140.20, 139.31, 138.89, 137.97, 136.69, 134.32, 131.30, 129.91, 129.52, 125.24, 121.65, 120.92, 116.59, 65.41, 53.18, 50.59, 30.30, 29.55 29.52, 29.48, 29.40, 29.32, 29.07, 29.02, 26.39, 25.95, 21.52, 14.75, 14.61. MS *m*/*z*: [M–Cl]^+^ calcd for C_44_H_57_BF_2_N_5_O_2_Cl, 736.76; found, 736.57. Anal. calcd for C_44_H_57_BF_2_N_5_O_2_Cl: C 68.44, H 7.44, N 9.07; found: C 68.35, H 7.53, N 9.11.

*Preparation of EtP5*: To a solution of 1‐ethoxy‐4‐methoxybenzene (3.35 g, 20.0 mmol) and paraformaldehyde (0.75 g, 25.0 mmol) in 1,2‐dichloroethane (300 mL), boron trifluoride diethyl etherate [BF_3_·O(C_2_H_5_)_2_, 2.52 mL, 20.0 mmol] was added under nitrogen atmosphere at 25 °C. Then, the mixture was stirred for 4 h. The solution was washed by saturated sodium chloride solution and dried by anhydrous sodium sulfate. The solvent was removed and the residue was purified by flash column chromatography on silica gel with CH_2_Cl_2_ as eluent, affording EtP5 as a white solid with 46% yield. ^1^H NMR (400 MHz, CDCl_3_, 25 °C, δ): 6.81 (s, 10H), 3.89 (m, 20H), 3.77 (s, 10H), 1.36 (m, 30H); ^13^C NMR (400 MHz, CDCl_3_, 25 °C, δ): 149.83, 128.54, 114.70, 63.67, 40.24, 31.71, 29.71, 22.77, 15.35, 14.24. MS *m*/*z*: [M^+^] calcd for C_55_H_70_O_10_, 891.14; found, 890.76. Anal. calcd for C_55_H_70_O_10_: C 74.13, H 7.92; found: C 74.23, H 7.86.

*Preparation of [2]Rotaxane BC12P5 (**5**)*: A mixture of **3** (62.49 mg, 0.10 mmol) and EtP5 (0.37 g, 0.40 mmol) was stirred in CHCl_3_ (0.40 mL) at −6 °C for 2 h. Then dibutyltindilaurate (one drop) and 1‐isocyanato‐3,5‐dimethylbenzene (0.20 g, 1.3 mmol) were added. The mixture was further stirred for 3 h. The solvent was removed and the residue was purified by flash column chromatography on silica gel with (CH_2_Cl_2_/MeOH = 25/1, v/v) eluent to afford **5** as a red solid with 76% yield. ^1^H NMR (400 MHz, DMSO‐*d*_6_, 25 °C, δ): 9.12 (s, 1H), 7.93 (s, 1H), 7.60 (d, *J* = 8 Hz, 2H), 7.54 (d, *J* = 8 Hz, 3H), 7.15 (s, 2H), 6.84 (s, 5H), 6.79 (s 5H), 6.65 (s, 1H), 6.20 (s, 2H), 5.63 (s, 2H), 3.97 (m, 3H), 3.86 (m, 16H), 3.63 (m, 13H), 3.49 (m, 2H), 2.45 (s, 6H), 2.24 (s, 6H), 1.44–1.35 (m, 36H), 0.97 (m, 2H), 0.74 (m, 2H), 0.59 (m, 6H), 0.27 (m, 2H), −0.2 (m, 2H), −0.4 (m, 2H), −0.65 (m, 2H), −0.81 (m, 2H); ^13^C NMR (400 MHz, CDCl_3_, 25 °C, δ): 156.06, 150.60, 149.47, 142.85, 138.93, 136.10, 135.76, 133.69, 131.47, 130.32, 130.09, 129.15, 125.84, 125.26, 122.89, 121.79, 121.62, 116.90, 116.51, 114.79, 66.22, 65.30, 63.86, 52.07, 48.37, 31.28, 30.78, 30.50, 30.06, 29.85, 29.70, 29.40, 28.93, 28.88, 27.12, 26.57, 25.73, 21.54, 15.74, 15.58, 14.76, 14.69. MS *m*/*z*: [M–Cl]^+^ calcd for C_99_H_127_BF_2_N_5_O_12_Cl, 1627.90; found, 1627.95. Anal. calcd for C_99_H_127_BF_2_N_5_O_12_Cl: C 71.49, H 7.70, N 4.21; found: C 71.41, H 7.78, N 4.26.

## Supporting information

As a service to our authors and readers, this journal provides supporting information supplied by the authors. Such materials are peer reviewed and may be re‐organized for online delivery, but are not copy‐edited or typeset. Technical support issues arising from supporting information (other than missing files) should be addressed to the authors.

Supplementary
