# Supplementary material for: Multistimuli Sensitive Behavior of Novel Bodipy‐Involved Pillar[5]arene‐Based Fluorescent [2]Rotaxane and Its Supramolecular Gel
Source: Adv Sci (Weinh). 2015 May 26;2(9):1500082. doi: 10.1002/advs.201500082 (PMC5115378; doi:10.1002/advs.201500082)
Supplement: Supplementary file 1 — Supplementary [file ADVS-2-0j-s001.pdf]

Copyright WILEY-VCH Verlag GmbH & Co. KGaA, 69469 Weinheim, Germany, 2015.

ADVANCED SCIENCE
Open Access

Supporting Information

for *Adv. Sci.*, DOI: 10.1002/advs. 201500082

Multistimuli Sensitive Behavior of Novel Bodipy-Involved Pillar[5]arene-Based Fluorescent [2]Rotaxane and Its Supramolecular Gel

Nana Sun, Xin Xiao, Wenjun Li, and Jianzhuang Jiang**

Copyright WILEY-VCH Verlag GmbH & Co. KGaA, 69469 Weinheim, Germany, 2013.

Supporting Information

Multi-Stimuli Sensitive Behavior of Novel Bodipy-containing Pillar[5]arene-based Fluorescent [2]Rotaxane and Its Supramolecular Gel

*Nana Sun, Xin Xiao, * Wenjun Li, and Jianzhuang Jiang **

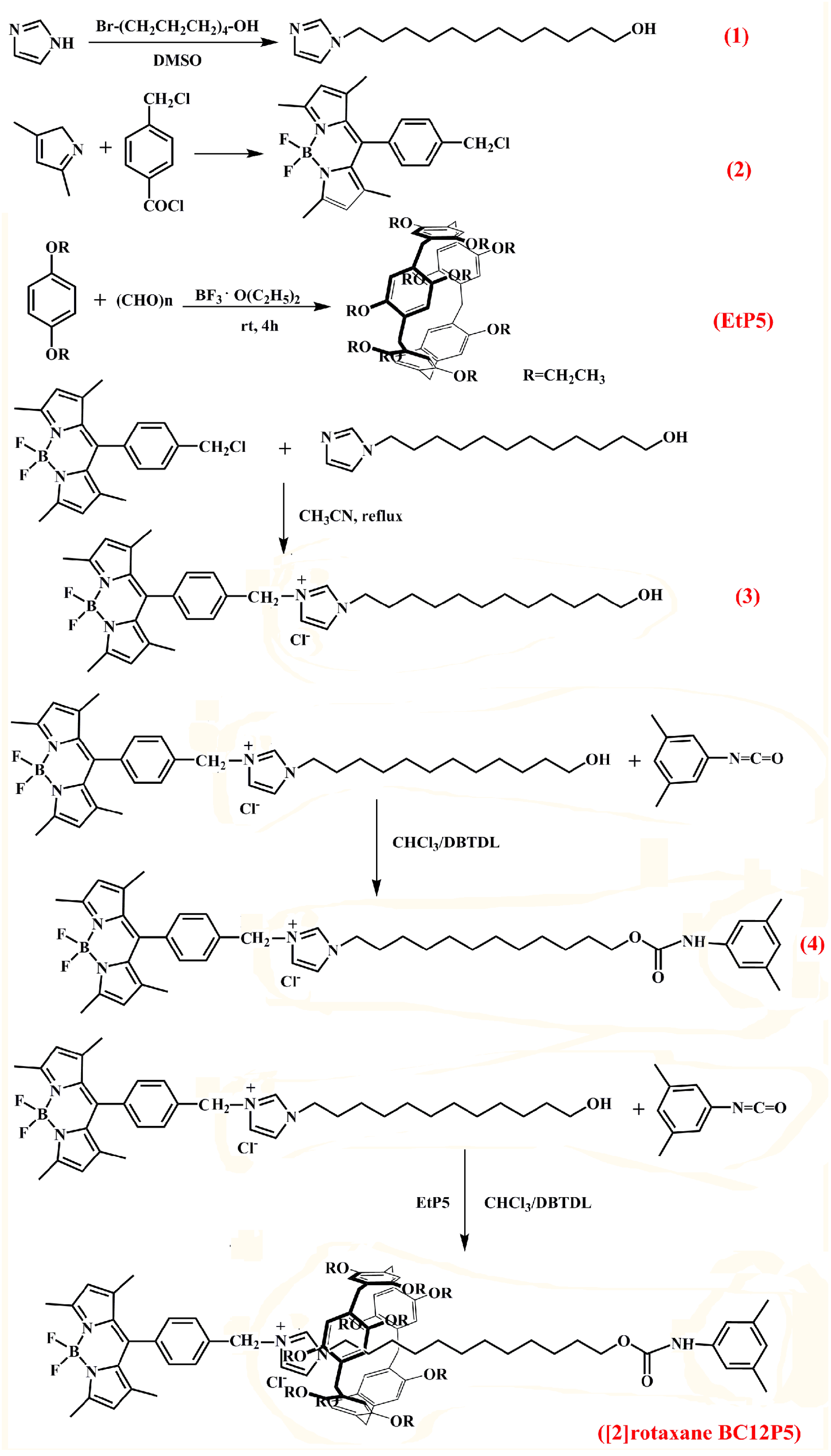

Scheme S1. Synthesis of [2]rotaxane BC12P5.

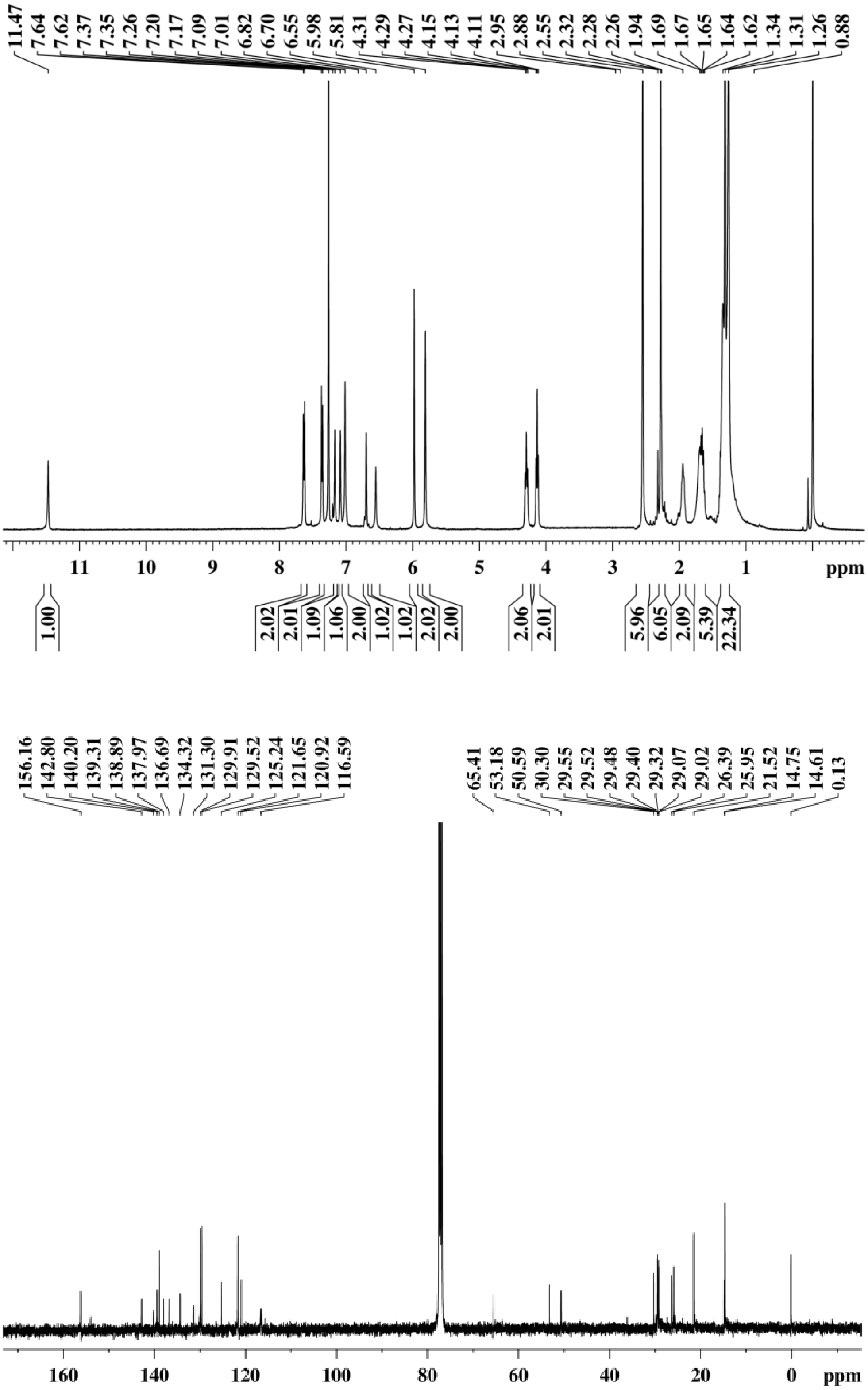

Figure S1. ^{1}H, ^{13}C NMR spectra of **4** recorded in $CDCl_3$ at 25 °C.

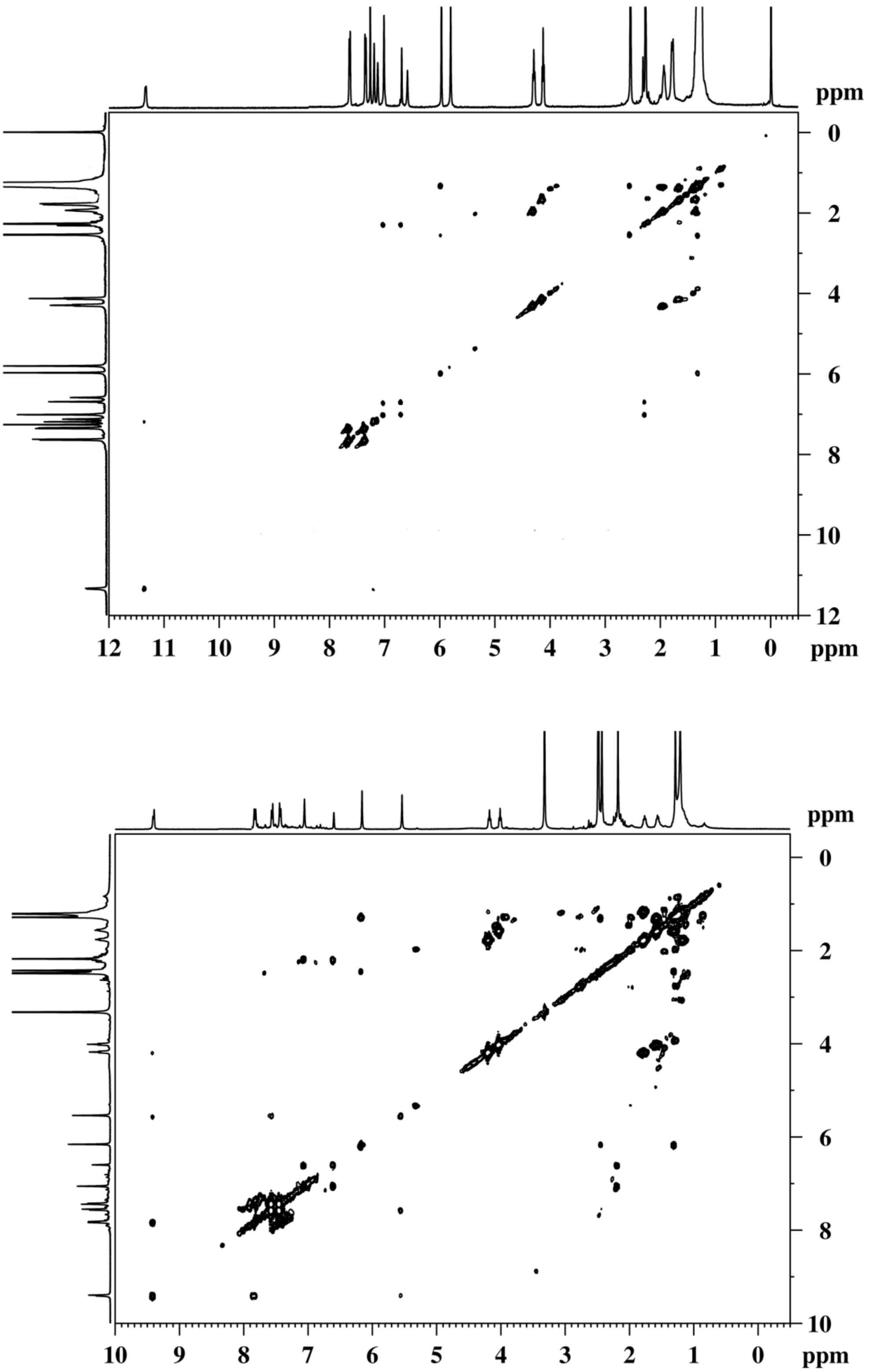

Figure S2. ^1H-^1H COSY NMR spectra of **4** recorded in $CDCl_3$ (top) and DMSO-d_6 (bottom) at 25 °C.

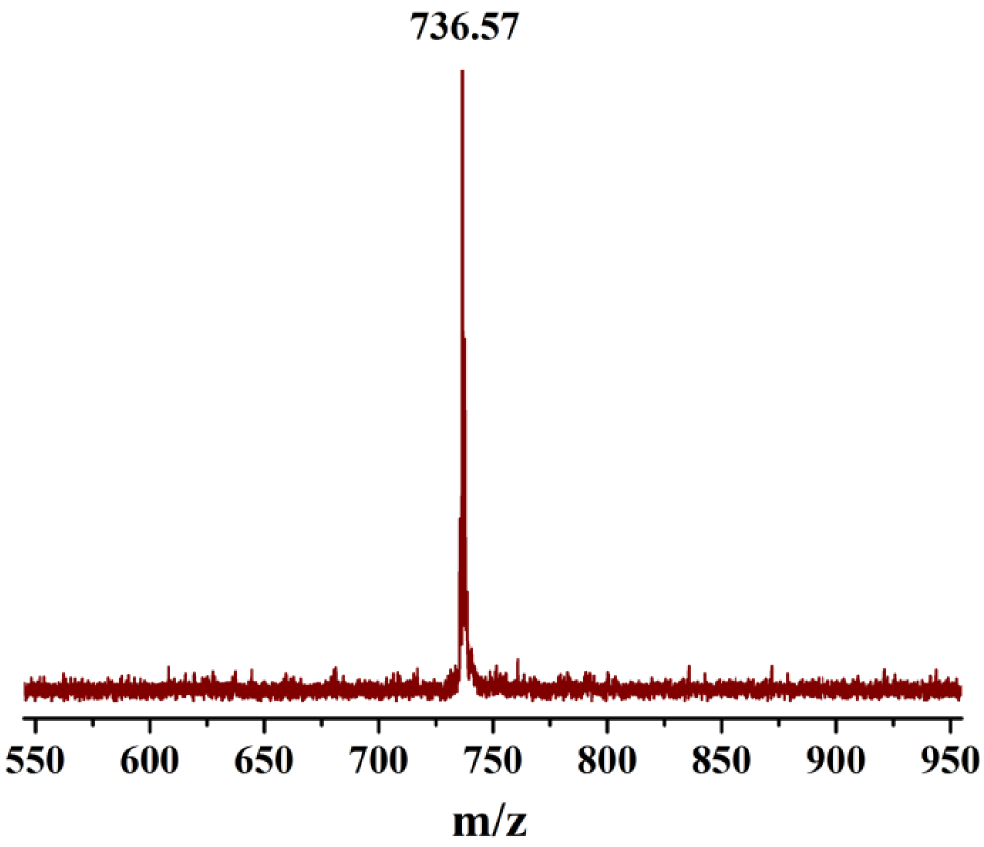

Figure S3. The MALDI-TOF mass spectrum of **4**.

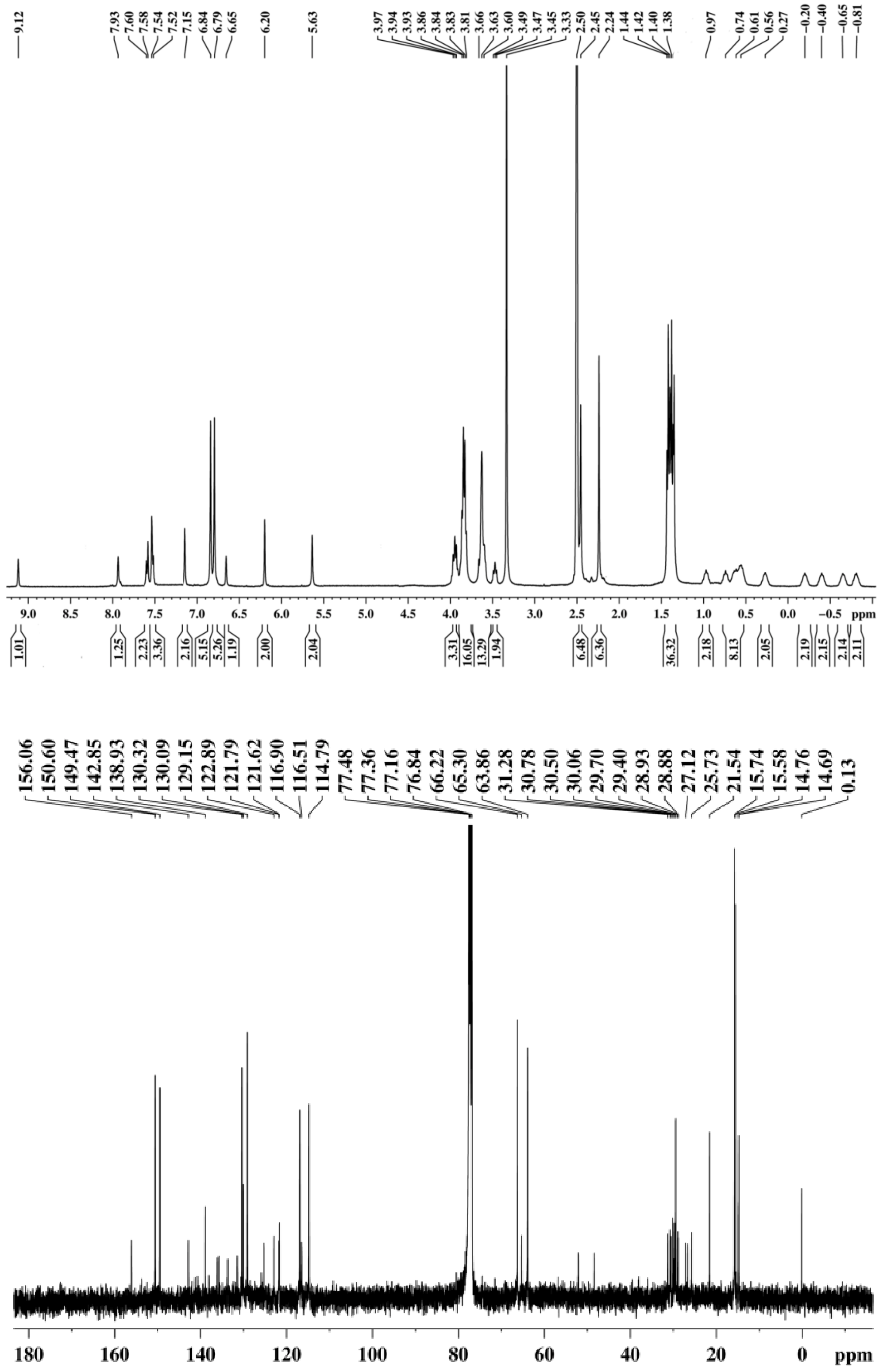

Figure S4. ^{1}H, ^{13}C NMR spectra of [2]rotaxane BC12P5 recorded in DMSO-d_6 at 25 °C.

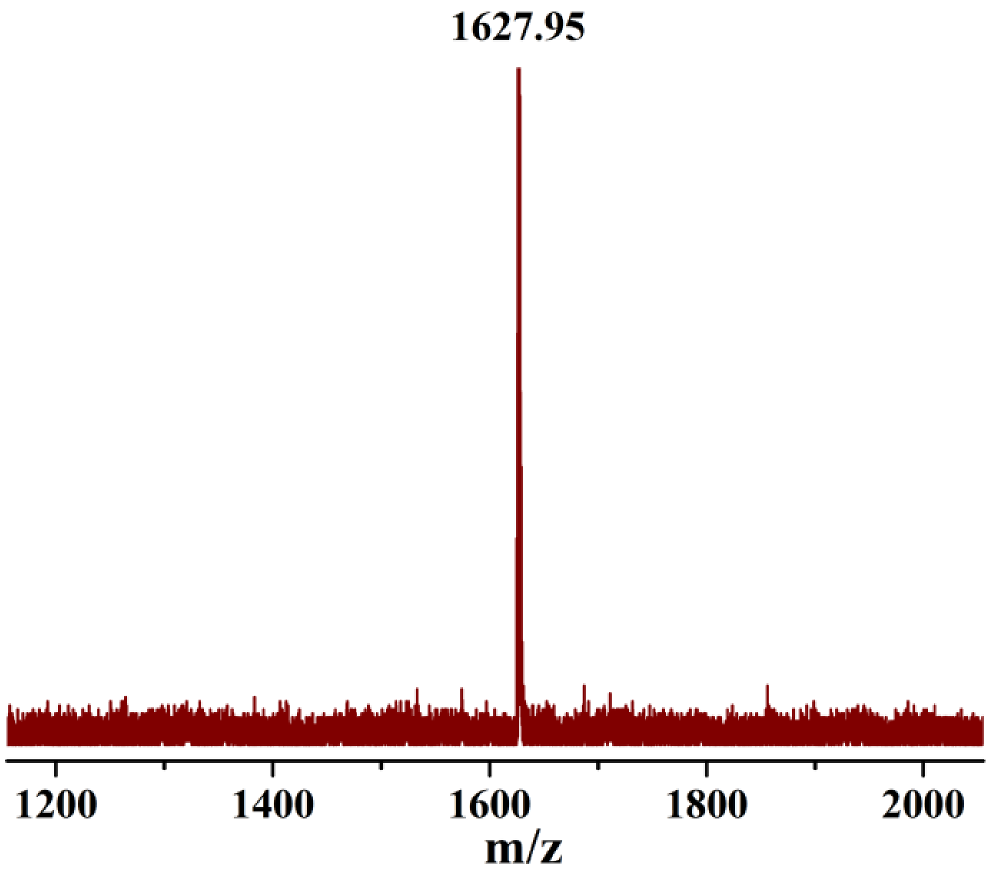

Figure S5. The MALDI-TOF mass spectrum of [2]rotaxane BC12P5.

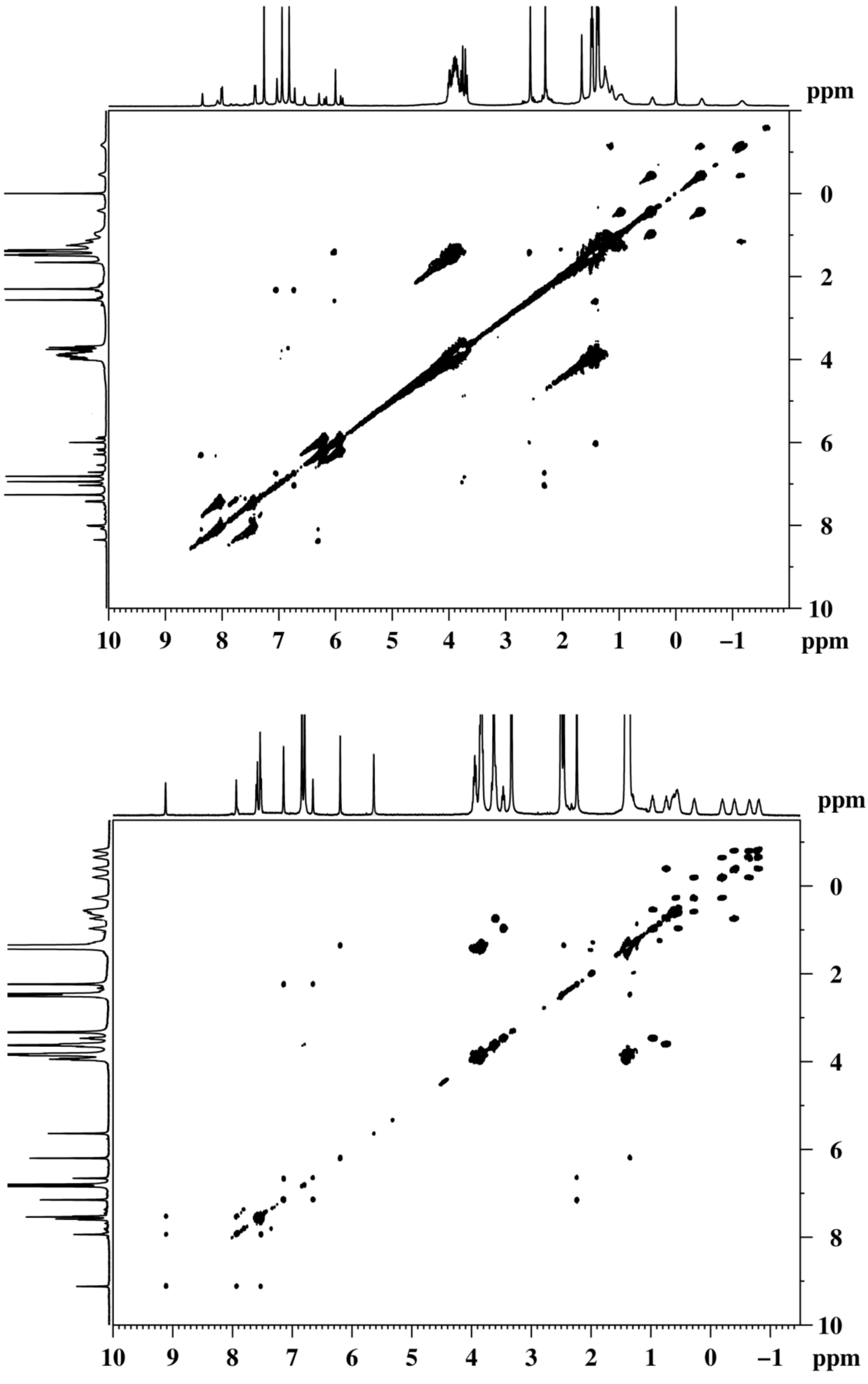

Figure S6. ^{1}H-^{1}H COSY NMR spectrum of [2]rotaxane BC12P5 recorded in $CDCl_3$ (top) and DMSO-d_6 (bottom) at 25 °C.

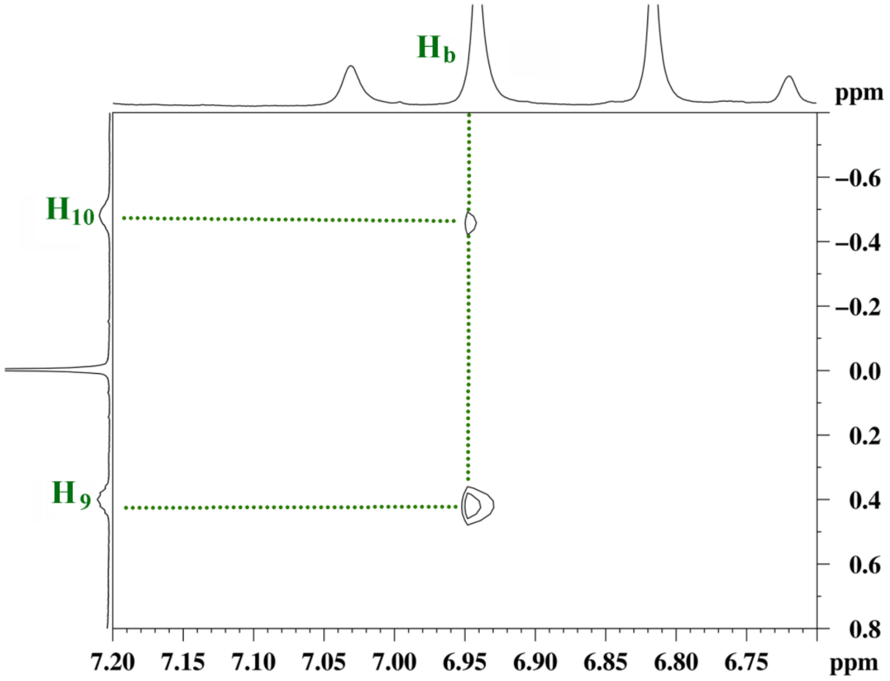

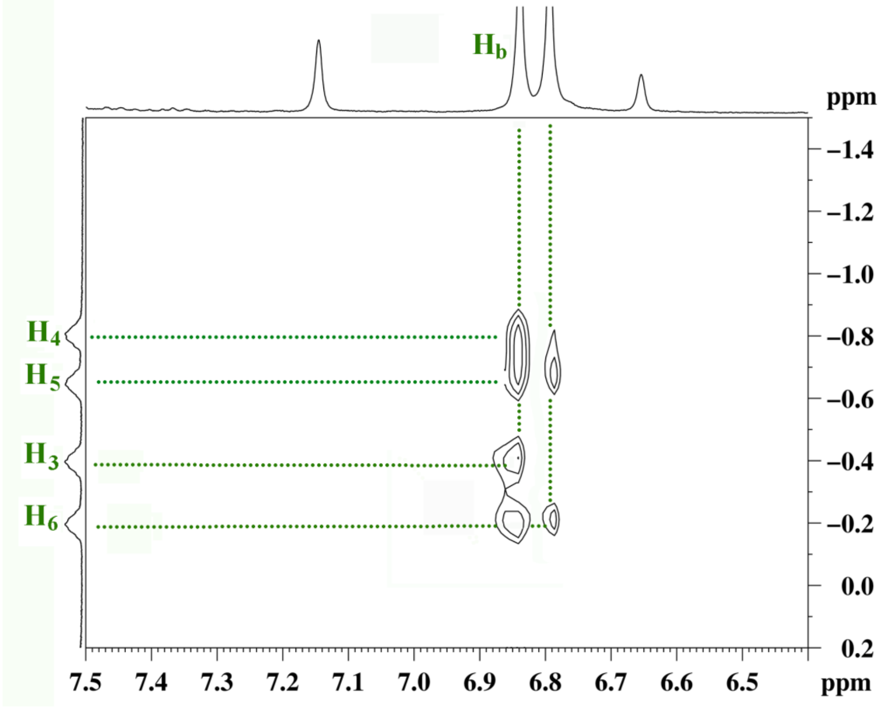

Figure S7. Partial NOESY NMR spectrum of [2]rotaxane BC12P5 recorded in $CDCl_3$ (top) and DMSO-d_6 (bottom) at 25 °C.

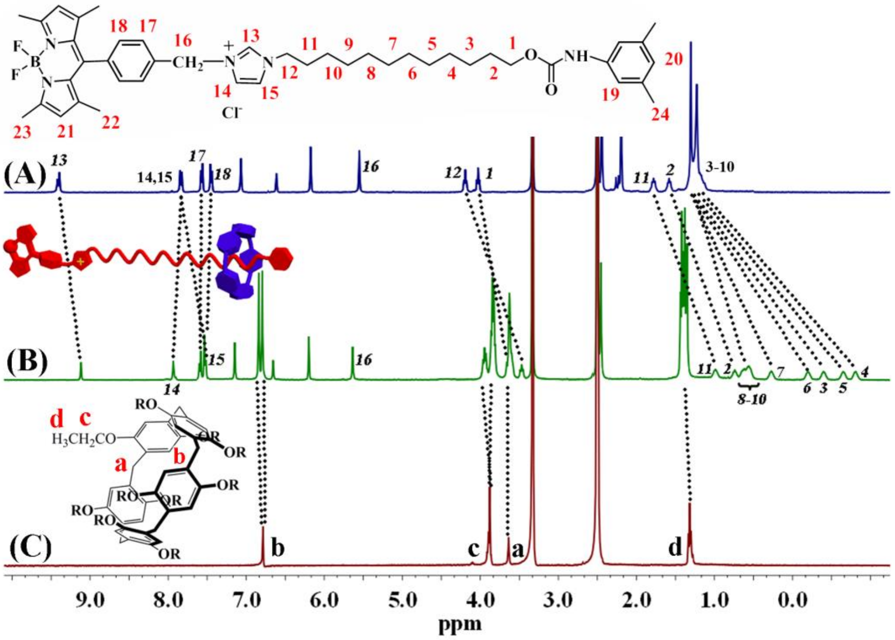

Figure S8. ^{1}H NMR spectra of compound **4** (A), [2]rotaxane BC12P5 (B), EtP5 (C) recorded in DMSO-d_6 at 25 °C.

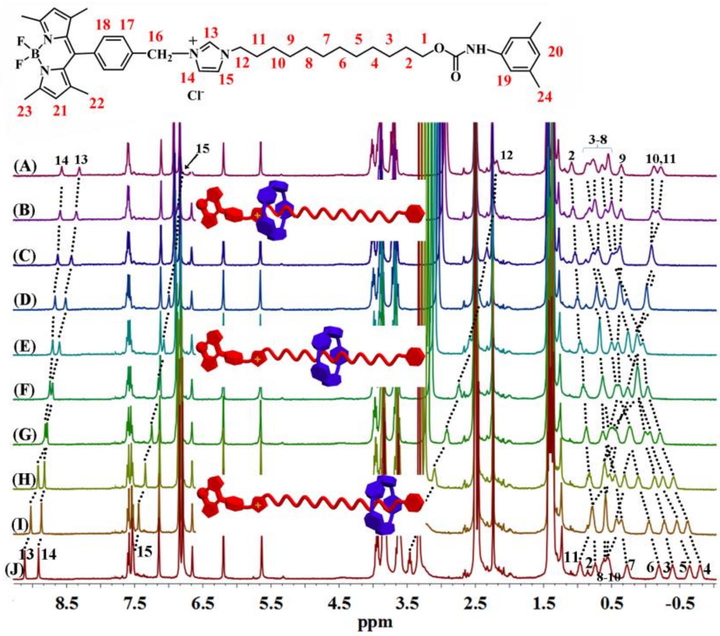

Figure S9. Systematic change in the ^{1}H NMR spectrum of [2]rotaxane BC12P5 along with the change in temperature: (A) 115 °C, (B) 105 °C, (C) 95 °C, (D) 85 °C, (E) 75 °C, (F) 65 °C, (G) 55 °C, (H) 45 °C, (I) 35 °C, and (J) 25 °C recorded in DMSO-d_6.

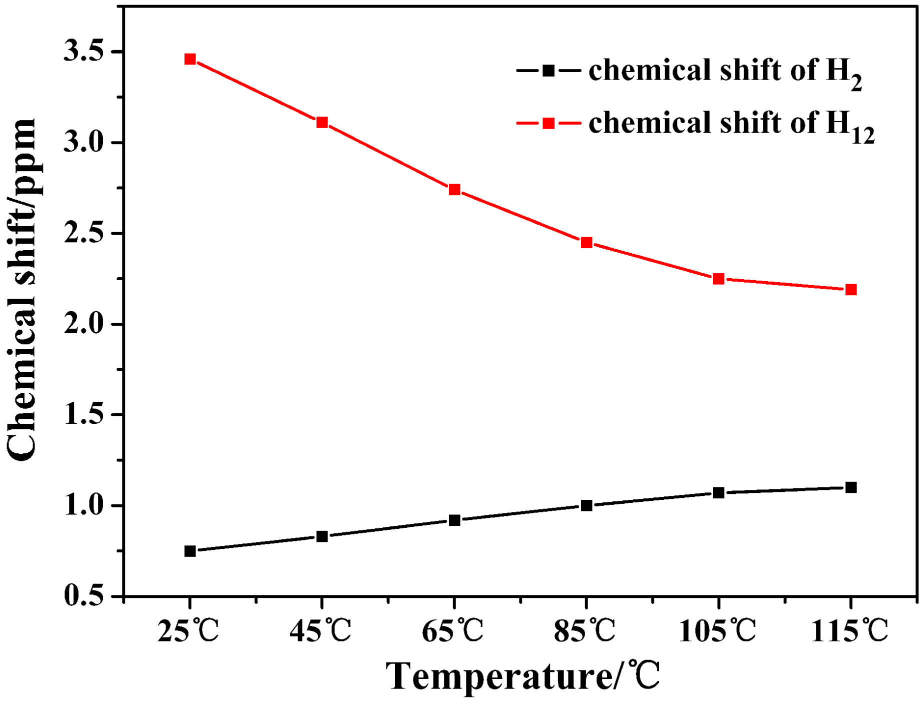

Figure S10. Temperature-dependent chemical shifts of H_2 and H_{12} for [2]rotaxane BC12P5 in DMSO-d_6.

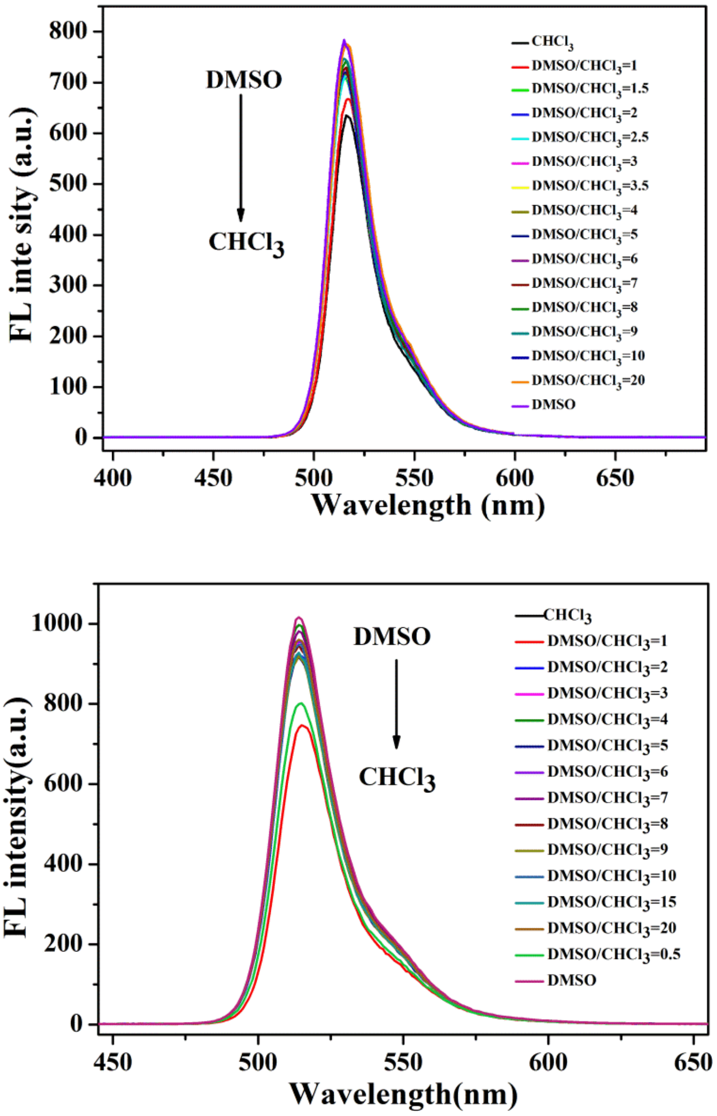

Figure S11. Systematic change in the fluorescence spectrum of [2]rotaxane BC12P5 (top) and compound **4** (bottom) along with change in the ratio of $CDCl_3$/DMSO-d_6 (v/v) recorded at 25 °C.

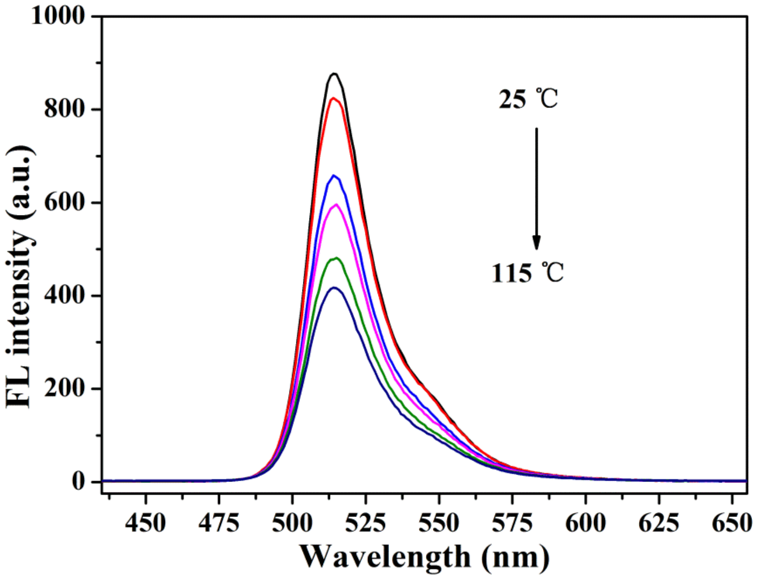

Figure S12. Systematic change in the fluorescence spectrum of compound **4** in DMSO (1×10^{-5} mol/L) along with the temperature change from 25 °C to 115 °C.

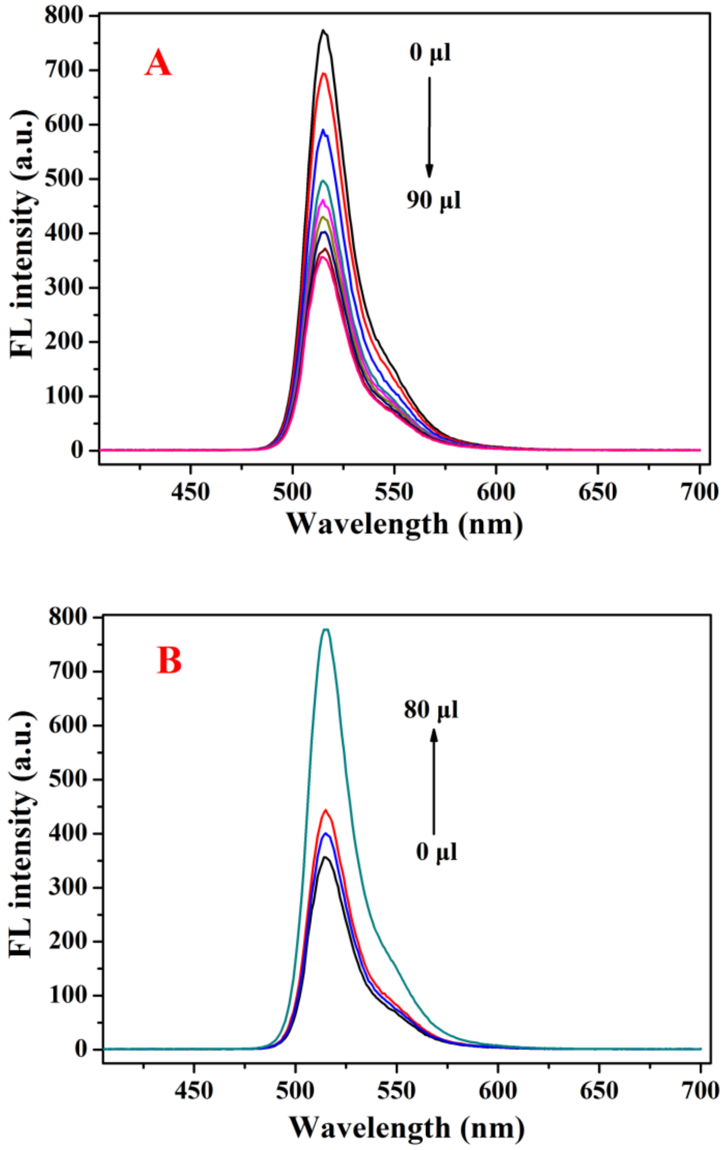

Figure 13. The fluorescence emission spectra of compound **4** (1×10^{-5}mol/L) in $CHCl_3$ upon addition of increasing amount of TEA (A) and then of increasing amount of TFA (B) at 25 °C.

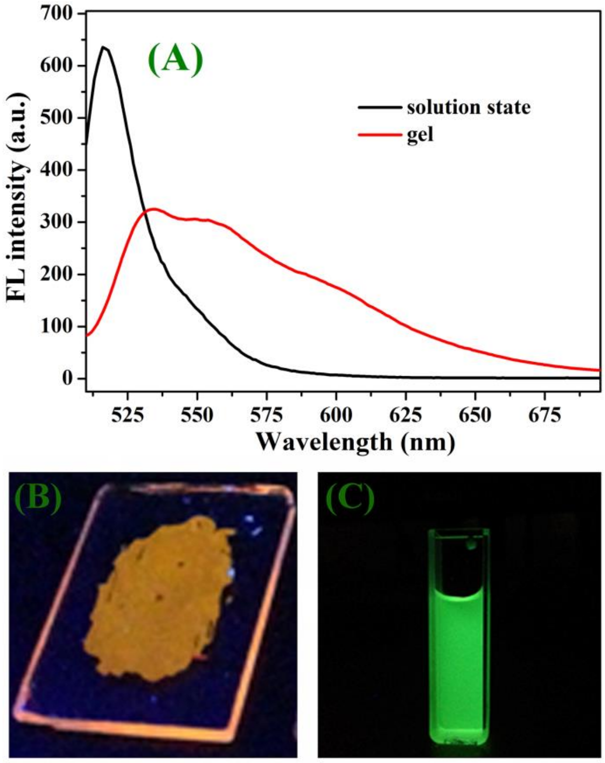

Figure S14. Fluorescence spectrum of [2]rotaxane BC12P5 in $CHCl_3$ solution (dark line) and gel (red line) recorded at 25 °C (A), photographs of gel (B) and solution of [2]rotaxane BC12P5 (C) under illuminated at 365 nm.

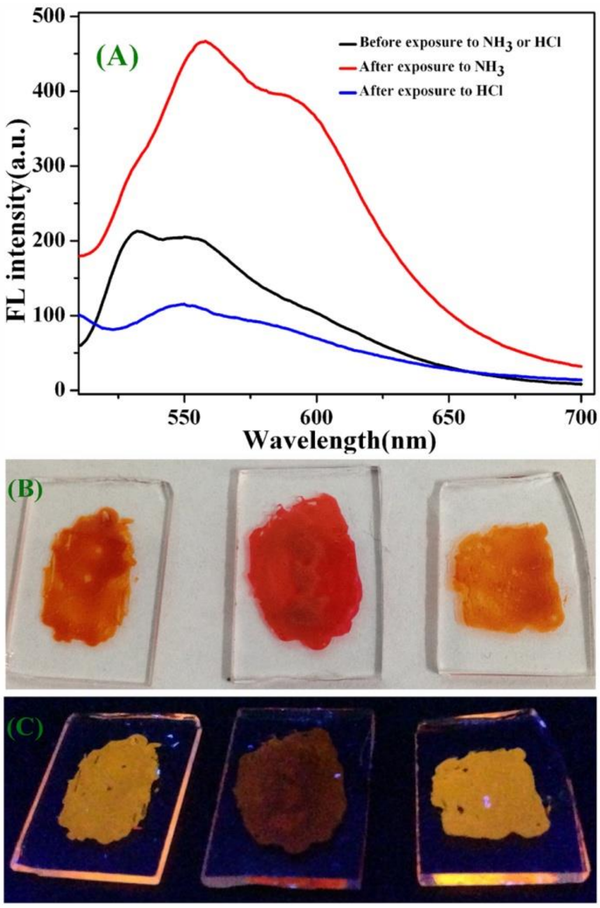

Figure S15. Change in the fluorescence spectrum of gel, before and after exposure to HCl/NH_3 for 10 min (A), photographs of gel before (left) and after exposure to HCl (middle), NH_3 (right) under ambient light (B) and illuminated at 365 nm (C).

Table S1. Chemical shifts of the protons of compound **4** and those in the [2]rotaxane BC12P5 (**5**) system in $CDCl_3$.

**Compound**	**δ (ppm)**										
	1	8	9	10	11	12	13	14	15	16	16′
**4**	4.14	1.31	1.31	1.31	1.96	4.29	11.47	7.18	7.09	5.81	5.81
**5**	3.85	0.95	0.39	-0.46	-1.19	3.98	8.39	8.09	6.30	5.90	6.21

Table S2. Chemical shifts of the protons of compound **4** and those in the [2]rotaxane BC12P5 (**5**) system in DMSO-d_6.

**complex**	**δ (ppm)**												
	1	2	3	4	5	6	7	8-10	11	12	13	14	15
**4**	4.03	1.58	1.27	1.27	1.27	1.27	1.27	1.27	1.78	4.19	9.42	7.83	7.83
**5**	3.65	0.74	-0.40	-0.81	-0.64	-0.20	0.28	0.58	0.98	3.46	9.11	7.96	7.51

Table S3. Chemical shifts of the protons on the axle of [2]rotaxane BC12P5 in solvents with different polarities ($CDCl_3$/DMSO-d_6, v/v).

$CDCl_3$/ DMSO-d_6	δ (ppm)													
	2	3	4	5	6	7	8	9	10	11	12	13	14	15
**DMSO-d_6**	0.75	-0.40	-0.80	-0.63	-0.20	0.27	0.60	0.60	0.60	0.97	3.46	9.11	7.94	7.52
**1/20**	0.75	-0.34	-0.69	-0.50	-0.07	0.34	0.60	0.60	0.45	0.79	3.35	9.04	7.94	7.46
**1/9**	0.76	-0.25	-0.52	-0.31	0.09	0.46	0.60	0.60	0.34	0.60	3.10	8.93	7.94	7.34
**1/7**	0.79	-0.20	-0.44	-0.20	0.18	0.52	0.66	0.51	0.27	0.48	2.99	8.89	7.94	7.28
**1/5**	0.84	-0.07	-0.29	-0.07	0.32	0.58	0.67	0.51	0.20	0.30	2.80	8.78	7.91	7.20
**1/3**	0.92	0.24	0.02	-0.15	0.52	0.73	0.73	0.45	-0.02	-0.02	2.38	8.58	7.89	6.99
**1/1**	1.06	0.97	0.97	0.97	0.97	0.97	0.84	0.36	-0.46	-1.19	1.09	7.64	7.71	6.27
**$CDCl_3$**	1.24	1.24	1.24	1.24	1.24	1.24	0.92	0.40	-0.47	-1.21	1.03	8.38	8.08	6.29

Table S4. Chemical shifts of the protons on the axle of [2]rotaxane BC12P5 in DMSO-d_6 at variable temperature.

Tempera-ture (℃)	δ (ppm)													
	2	3	4	5	6	7	8	9	10	11	12	13	14	15
**25**	0.75	-0.40	-0.80	-0.63	-0.20	0.27	0.58	0.58	0.58	0.97	3.46	9.11	7.94	7.52
**45**	0.83	-0.13	-0.41	-0.25	0.10	0.45	0.58	0.61	0.31	0.51	3.11	8.92	7.91	7.34
**65**	0.92	0.12	-0.03	0.12	0.40	0.64	0.56	0.42	0.12	0.27	2.74	8.72	7.87	7.16
**85**	1.00	0.38	0.27	0.38	0.59	0.72	0.56	0.39	-0.01	0.01	2.45	8.52	7.83	7.00
**105**	1.07	0.50	0.58	0.50	0.74	0.77	0.56	0.38	-0.11	-0.18	2.25	8.35	7.78	6.86
**115**	1.10	0.74	0.65	0.54	0.77	0.81	0.55	0.36	-0.11	-0.24	2.19	8.32	7.75	6.82
